# The Cryptochrome CryA Regulates Lipid Droplet Accumulation, Conidiation, and Trap Formation via Responses to Light in *Arthrobotrys oligospora*

**DOI:** 10.3390/jof10090626

**Published:** 2024-09-01

**Authors:** Yanmei Shen, Xuewei Yang, Meichen Zhu, Shipeng Duan, Qianqian Liu, Jinkui Yang

**Affiliations:** 1 State Key Laboratory for Conservation and Utilization of Bio-Resources, Yunnan University, Kunming 650091, China; shenyanmei@stu.ynu.edu.cn (Y.S.); yangxuewei@stu.ynu.edu.cn (X.Y.); zmc201789@163.com (M.Z.); duanshipeng@stu.ynu.edu.cn (S.D.); liuqianqian614@163.com (Q.L.); 2 Key Laboratory for Microbial Resources of the Ministry of Education, Yunnan University, Kunming 650091, China; 3 School of Life Sciences, Yunnan University, Kunming 650091, China

**Keywords:** nematode-trapping fungi, light regulation, mycelial development, secondary metabolism, pathogenicity

## Abstract

Light is a key environmental factor affecting conidiation in filamentous fungi. The cryptochrome/photolyase CryA, a blue-light receptor, is involved in fungal development. In the present study, a homologous CryA (AoCryA) was identified from the widely occurring nematode-trapping (NT) fungus *Arthrobotrys oligospora*, and its roles in the mycelial growth and development of *A. oligospora* were characterized using gene knockout, phenotypic comparison, staining technique, and metabolome analysis. The inactivation of *AocryA* caused a substantial decrease in spore yields in dark conditions but did not affect spore yields in the wild-type (WT) and ∆*AocryA* mutant strains in light conditions. Corresponding to the decrease in spore production, the transcription of sporulation-related genes was also significantly downregulated in dark conditions. Contrarily, the ∆*AocryA* mutants showed a substantial increase in trap formation in dark conditions, while the trap production and nematode-trapping abilities of the WT and mutant strains significantly decreased in light conditions. In addition, lipid droplet accumulation increased in the ∆*AocryA* mutant in dark conditions, and the mutants showed an increased tolerance to sorbitol, while light contributed to the synthesis of carotenoids. Finally, AoCryA was found to affect secondary metabolic processes. These results reveal, for the first time, the function of a homologous cryptochrome in NT fungi.

## 1. Introduction

Asexual spore formation is important for the growth, development, and pathogenicity of filamentous fungi [1]. The ubiquitous *Aspergillus fumigatus* produces large numbers of conidia that are readily airborne [2]. Inhalation of airborne spores by humans can lead to severe invasive pulmonary aspergillosis, which occurs mainly in immunocompromised individuals and has a mortality rate exceeding 50% [3,4]. Conidia of this pathogenic fungus also contain potent allergens that can trigger allergic bronchopulmonary aspergillosis in susceptible individuals [5,6]. Similarly, *Magnaporthe oryzae* causes rice blast, the most devastating disease to affect cultivated rice. The fungus spreads via conidia, with infection initiated by the germination of spores on leaf surfaces and the subsequent formation of appressoria to invade rice tissues [7,8]. Another example is the nematode-trapping (NT) fungus *Arthrobotys flagrans*, which forms sticky three-dimensional networks to predate nematodes. Notably, it produces resistant chlamydospores that survive passage through the gastrointestinal tract and then germinate in feces, thereby reducing nematode populations in the feces of ruminants. *A. flagrans* has proven effective as a nematode control agent for horses, cattle, and other animals [9,10,11].

Conidia formation is a complicated process that can be categorized into a number of distinct phases [12,13]. For decades, *Aspergillus nidulans* has been utilized as a model species to study conidiation, resulting in substantial insights into the regulatory pathways involved [14,15,16]. In *A. nidulans*, numerous genes are involved in the regulation of conidiation, including various upstream activators, central regulators, and light-responsive and velvet regulators [17,18]. The initiation of conidiation is modulated by upstream developmental activators, which contain FlbA, FlbB/D/E, and FlbC. FluG operates upstream of FlbB/D/E and FlbC, which activate the FLB module [19]. Additionally, the regulatory framework encompasses central regulatory pathways and velvet family proteins, as well as heterotrimeric G protein and Ras-mediated signaling pathways [16]. BrlA, AbaA, and WetA are all crucial regulators of conidiation in species of *Aspergillus* and several filamentous fungi; they have been shown to orchestrate conidial initiation, elongation, and termination [20]. The spore-producing mechanism of fungi is broadly regulated; its development is mediated by light and is dependent on a sophisticated genetic repertoire [21,22].

Light controls many processes in filamentous fungi. *A. nidulans* develops asexually when grown in light but forms complex sexual zoosperms when grown in darkness [23]. In *A. nidulans*, asexual spore formation occurs under red (680 nm) and blue (450 nm) light, but the process is less pronounced under white light [24]. *Neurospora crassa* is one of the most widely used fungi in studies of light response because of the key role played by light-regulating factors in the manipulation of its circadian rhythms [25,26]. Two of its proteins have been identified as major regulators of blue-light sensing; these are termed white collar 1 (WC-1) and white collar 2 (WC-2) [27]. Additionally, photolyase has been recognized as a key protein in ultraviolet radiation and blue-light sensing, as it exhibits both photo-dissociative activity and the ability to complement the DNA repair mechanism [28]. Together with cryptochrome photoreceptors, the above proteins form a family (cryptochrome/photokinase family, CPF). One recently revealed CPF is composed of DASH (Drosophila, *Arabidopsis thaliana*, *Polycystis aeruginosa*, and human)-type cryptochrome (cry-DASH); it has been found in bacteria, plants, and animals, as well as in fungi [29]. Cry-DASH proteins are involved in the regulation of photo-development in *Sclerotinia sclerotiorum* and *Fusarium fujikuroi* [30], as well as pigment accumulation in *Fusarium verticillioides* [31]. Cryptochrome is an important component of the circadian clock in mammals [32], and it has been shown to act as a magnetic receptor in migratory birds [33]. The photolyase/cryptochrome CryA senses blue light and ultraviolet radiation A, and fungal development is regulated by the interaction of various photoreceptors [28,34]. Thus, the photoreceptor cryptochrome CryA plays a crucial role in fungal development. Though fungal light responses have been widely studied for over 50 years, the advent of new experimental methods, such as genome-wide expression analysis and whole genome sequencing, has resulted in deeper insights into fungal photoreceptors and signaling pathways [24,35,36,37,38,39].

NT fungi are a unique class of ascomycete fungi that parasitize, catch, colonize, and paralyze nematodes. These fungi are natural predators of nematodes, and they play a vital role in the biocontrol of plant–parasitic nematodes [40]. *Arthrobotrys oligospora*, a widely occurring NT fungus, captures nematodes by forming traps specialized in trophic hyphae [41,42]. This group of fungi can be regarded as a crucial factor in regulating nematode communities in nature, with the formation of conidia and traps being vital traits for the effectiveness of biocontrol preparations [43,44,45]. Recently, the three central regulatory genes AoWetA, AoBrlA, and AoAbaA were shown to be essential for conidia and trap formation as well as for regulating mycelial development, autophagy, and secondary metabolism [46]. However, the function of light signaling in NT fungi remains largely unexplored, particularly with regard to its regulation of conidia and trap formation. In the present study, we aim to explore the role of light regulation in NT fungi, and a homologous CryA (AoCryA) was identified in *A. oligospora* through gene knockout, phenotypic analysis, and metabolomic analysis.

## 2. Materials and Methods

### 2.1. Strains and Culture Conditions

A wild-type (WT) strain of *A. oligospora* (ATCC24927) and knockout strains were routinely maintained on a PDA medium (1 L: potato 200 g, glucose 20 g, and agar 20 g). The plasmids pRS426 and pCSN44 were preserved in *Escherichia coli* and used to construct recombinational plasmids using the homologous recombination function of *Saccharomyces cerevisiae* FY834. In addition, SC-Ura medium was used to screen single colonies of yeast with fusion plasmid [47]. YPD medium (1 L: yeast extract 10 g, peptone 20 g, and agar 5 g) was used to culture the yeast. A PDAS medium was used for the cultivation of transformants [48]. Water agar (WA) medium was used for trap induction under nutrient-deprived conditions, and *Caenorhabditis elegans* was cultured at room temperature in a sterile oatmeal medium. WT and knockout strains were cultivated at 28 °C, while the yeast was cultivated at 30 °C. For white-light experiments, plates were incubated for 5 to 7 days at 28 °C, either in the dark or under light emitted by light-emitting diode (LED) arrays with an intensity of 6 to 10 μmol photons/m^−2^/s, and blue light experiments were undertaken under LED lamps with wavelengths of 455–470 nm [49]. The map information of plasmids pRS426 and pCSN44 is described in Figure S1.

### 2.2. Bioinformatic Analysis

According to the CryA proteins of the model fungi *N. crassa* (XP_964834) and *A. nidulans* (XP_657991), a homologous protein was identified from *A. oligospora* (AoCryA, AOL_s00215g193). The pI/MW of *AocryA* was calculated via online software ExPASy-ProtParam (https://web.expasy.org/protparam/), and analysis of conserved structural domains was carried out using the InterProScan program (https://www.ebi.ac.uk/interpro/). CryA homologs were blasted from diverse fungi, and sequence similarities between *AocryA* and other homologs were compared using DNAman software (version 6). Homologous CryA proteins were aligned with Clustalx, and neighbor-joining trees were built using MEGA 6 software [50].

### 2.3. The AocryA Knockout

Replacement of the target gene with the hygromycin-resistant gene *hph* was achieved through homologous recombination [47]. The sequence of the target gene was downloaded from the NCBI database, and the 5′ and 3′ homologous arms—of approximately 2000 bp—were amplified using specific primers (Table S1). The recombinational plasmids were constructed with pRS426 as a vector backbone using the homologous recombination ability of yeast FY834, and the replaced fragments were inserted into the protoplasts of *A. oligospora* using chemical conversion. After the transformants were cultured, single colonies with the correctly replaced target gene were selected on a PDAS medium (1 L: potato 200 g, glucose 20 g, molasses 5 g, sucrose 205 g, agar 5 g) containing hygromycin B (200 μg/mL) [46]. Finally, these transformants were further validated using PCR and real-time quantitative PCR (RT-PCR) analysis [51]. The primers for gene disruption and verification are listed in Table S1.

### 2.4. Determination of Mycelial Growth, Spore Yield, and Germination Rate

We synchronized the growth of knockout and WT strains on a PDA plate for 5–7 days. Subsequently, pieces 7 mm in diameter were incubated onto PDA, TG (1 L: glucose 10 g, peptone 10 g, and agar 5 g), and TYGA (1 L: peptone 20 g, yeast extract 10 g, glucose 20 g, molasses 5 g, and agar 5 g) media at 28 °C, and colony diameters were measured daily over a 1–5 day period. To determine spore yield, the fungal strains were cultured on a CMY (1 L: corn 10 g, yeast extract 5 g, and agar 20 g) medium at 28 °C for 10–14 days. Spores were then quantified as previously described [48]. Then, approximately 20,000 spores were spread uniformly on MM medium, and the conidial germination rate was quantitated at 4, 8, and 12 h incubation [52]. All phenotypic analyses were performed in triplicate.

### 2.5. Observation of Mycelial Septa, Nuclei, Lipid Droplets (LDs), and Endocytosis

The mycelium was cultured on a PDA medium under light and dark conditions for 5 days. Mycelium was stained with Calcofluor White (CFW, Sigma-Aldrich, St. Louis, MO, USA) and 4′,6-diamidino-2-phenylindole (DAPI, Sigma-Aldrich) for 10 min. Mycelial septa and nuclei were counted under a fluorescence microscope. The LDs in the mycelial cells were treated with 10 μg/mL of boron dipyrrolidine dye (BODIPY, Sigma-Aldrich) for 25 min, and LDs were then observed using a fluorescence microscope. Endocytosis was labeled using an FM4-64 red fluorescent marker [53]. The time of entry of the dye into mycelial cells was also recorded. In addition, the fresh mycelia were treated and observed using transmission electron microscopy (TEM) (JEM-1400PLUS, Tokyo, Japan).

### 2.6. Analysis of Trap Formation, Pathogenicity, and Proteolytic Activity

The spore suspensions of the WT and knockout strains (approximately 20,000 spores each) were spread on a nutrient-poor medium WA (water agar) and cultured at 28 °C for 3 days. Then, approximately 200 nematodes were added to induce trap formation. Traps and captured nematodes were observed and recorded at 12, 24, 36, and 48 h post-induction. For the proteolytic activity assay, equal amounts of mycelial pieces were transformed into 100 mL of PDB broth and fermented at 28 °C with shaking at 180 rpm for 3 days. Subsequently, 8 mL of skim milk (3%) was added to each culture, and incubation continued under the same conditions for an additional 2 days. The cultures were then filtered to collect the supernate. Next, 20 mL of skim milk was added to 100 mL of the WA medium; this was mixed thoroughly and poured into 9 cm Petri dishes. Five wells (0.9 cm in diameter) were punched into each plate. Blank control (untreated PDA solution) was added to one well; the supernate from the WT strain and each of the mutant strains, respectively, was added to the remaining wells. The Petri dishes were maintained at 37 °C for 12–16 h. The sizes of the hydrolysis zones were photographed and recorded so that extracellular protease activity could be qualitatively assessed [54]. The above experiments were all performed in triplicate.

### 2.7. Stress Tolerance Analysis

The WT and knockout strains were synchronized and grown on PDA for 5 days; then, colonies of the same size were transferred to a TG plate (with the addition of chemical reagents) and incubated at 28 °C for 5 days. The diameter of each colony was recorded, and the relative growth inhibition rate (RGI) of fungal growth was then calculated [54].

### 2.8. RT-PCR Analysis

After the WT and knockout strains had grown on the CMY plates for 3, 5, and 7 days, mycelial samples were harvested, and total RNA was isolated using an RNA extraction kit (Axygen, Jiangsu, Suzhou, China). The *β*-*tubule* gene (*AOL_s0076g640*) was used as an internal reference [55], and RT-PCR was performed to analyze the transcription of genes involved in spore production and lipid metabolism using the primers listed in Table S1. The relative transcript level (RTL) of each gene was quantified using the 2^−ΔΔCT^ method, and each gene was analyzed in triplicate.

### 2.9. Secondary Metabolic Analysis

The WT and knockout strains were activated on a PDA plate for 5 days. The cultures were then inoculated into PDB broth at 28 °C and shaken at 180 rpm for 7 days. Next, the mycelia were filtrated and quantified, and an equal volume of ethyl acetate was used to extract the fermentation broth. The compounds were subsequently dissolved in chromatographically pure methanol. The resulting solutions were analyzed using LC-MS, and metabolic profiles were analyzed using Compound Discoverer 3.0. In addition, the peak areas were extracted to quantify the content of arthrobotrisins based on their characteristic ionic peaks at *m*/*z* 139, 393, and 429. Each sample was processed in triplicate for biological replication [56]. 

### 2.10. Data Analysis

All experimental data are expressed as mean ± standard deviation (SD) from three independent replicates. Statistical difference was evaluated using one-way ANOVA with Prism 9.0. *p >* 0.05 was considered to indicate a statistically significant difference.

## 3. Results

### 3.1. Bioinformatics Analysis of AoCryA and Verification of Deletion Strains

The CryA protein comprises 618 amino acids; it has an isoelectric point of 8.44 and a molecular weight of 71.0 kDa. Phylogenetic analysis reveals that the homologous proteins of CryA were separated into two groups, and these homologs from NT fungi were clustered into a single clade. Additionally, AoCryA shares a high degree of sequence similarity with homologs from different NT fungi, such as *Dactylellina haptotyla* (71.3%), *Arthrobotrys_entomopaga* (71.2%), and *A. flagrans* (69.9%). In addition, a domain structure analysis shows that CryA homologs from different fungi all contain a PhrB domain (Figure S2A).

Knockout strains were obtained by homologous recombination and validated by PCR and RT-qPCR methods. Three knockout strains (∆*AocryA-1*, ∆*AocryA-2*, and ∆*AocryA-3*) were obtained (Figure S2B,C).

### 3.2. AoCryA Is Involved in Sporulation by Regulating the Response of Light

**Figure 1:**
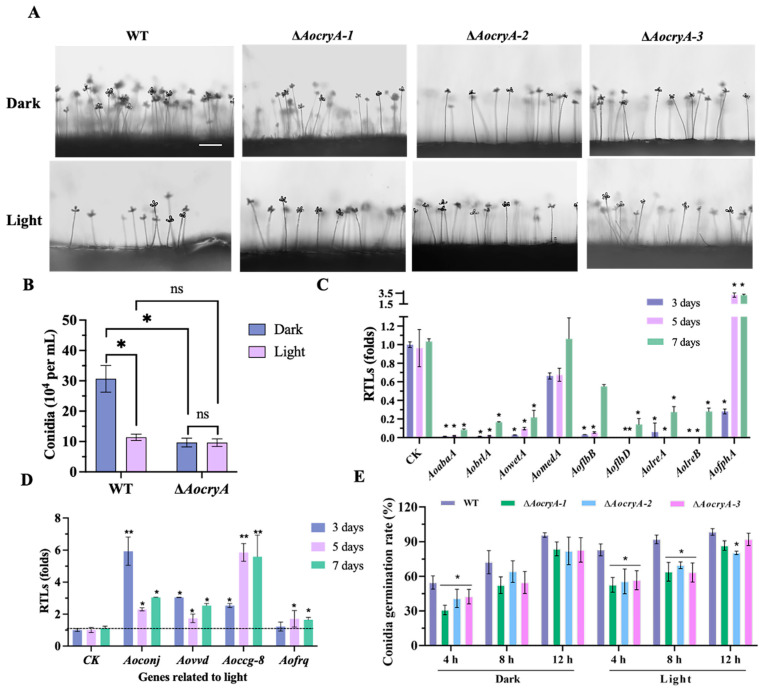
Observation of spore production-related traits. (**A**) Morphology of conidiophores after 3 days post-incubation on PDA medium. Bar = 100 µm. (**B**) Spore production after 14 days post-incubation on CMY medium. * *p* < 0.05 (**C**) Relative transcript levels (RTLs) of sporulation-related genes in the mutant strains versus WT under dark conditions. The expression of target genes in WT were served as control. * *p* < 0.05. (**D**) RTLs of light regulation-related genes in the mutant strains under the dark conditions. * *p* < 0.05, ** *p* < 0.01. The expressions of target genes in mutant strain under dark conditions served as control. (**E**) Germination rate of spores after incubation on MM medium for 4, 8, and 12 h. * *p* < 0.05.

Observations of conidiophores reveal that the mutant strains exhibited reduced numbers of conidiophores under dark conditions compared with the WT strain (Figure 1A). Quantitative analysis further confirms that the spore production in the mutant strains was only 30% of that of the WT strain under dark conditions (Figure 1B). In addition, the spore production of mutants was unaffected by light exposure. In contrast, the WT strain showed a substantial decrease in spore production of approximately 60% after exposure to light (Figure 1B). Furthermore, under dark conditions, the RTLs of sporulation-related genes, such as *AoabaA*, *AobrlA*, *AowetA*, *AoflbD*, *AolreA*, and *AolreB*, were substantially downregulated in the ∆*AocryA* strains, compared with WT strains, at 3, 5, and 7 days (Figure 1C). Given the reduced spore production of the WT strain under light conditions, we also analyzed the RTLs of light-regulated genes and found that these were remarkably upregulated under light conditions, compared with dark conditions (Figure 1D). In addition, the spore germination rate was delayed in the ∆*AocryA* strains. Specifically, under dark conditions, the germination rate of the ∆*AocryA* strains was remarkably reduced, compared with the WT strain at 4 h. Additionally, under light conditions, the germination rates of the mutants were reduced at both 4 and 8 h compared with the WT strain (Figure 1E).

### 3.3. AoCryA Affects the Length of Hyphal Cells and the Number of Nuclei

**Figure 2:**
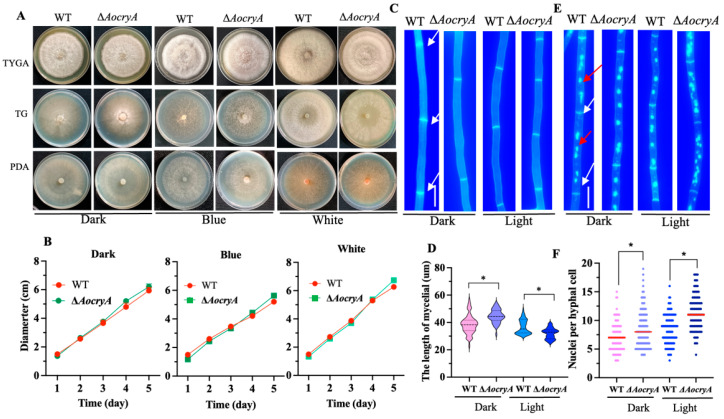
Mycelial growth rates, septa, and nuclei of the WT and the ∆*AocryA* strains. (**A**) The wild-type (WT) and the ∆*AocryA* strains were incubated on PDA, TYGA, and TG media under different light conditions at 28 °C for 5 days. (**B**) Comparison of colony growth rates on PDA medium under different light conditions. (**C**) Mycelial septa of WT and ∆*AocryA* strains under dark and light conditions. Scale bar—5 μm. White arrows indicate mycelial septa. (**D**) Mycelial cell lengths of WT and ∆*AocryA* strains. A total of 100 cells were randomly selected for measurement. * *p* < 0.05. (**E**) Observation of nuclei using 4′,6-diamidino-2-phenylindole (DAPI) staining under dark and light conditions. Scale bar—5 μm. White arrows indicate mycelial septa, and red arrows indicate nuclei. (**F**) Numbers of nuclei. A total of 100 cells were randomly selected for measurement. The red line shows the average value. * *p* < 0.05.

Under dark, white-light, and blue-light conditions, the WT and ∆*AocryA* strains exhibited no significant differences in mycelial growth after 5 days of incubation at 28 °C on TG, TYGA, and PDA media (Figure 2A,B and Figure S3). Following CFW staining, the mycelia of ∆*AocryA* mutants showed a reduction in cell septa and significant increases in cell length (20–25%) compared with the WT strain under dark conditions, whereas the mycelia of ∆*AocryA* mutants showed an increase in cell septa and remarkable reductions in cell length (7–15%) under light conditions (Figure 2C,D). Additionally, DAPI staining revealed an increased number of nuclei in the ∆*AocryA* strain under dark and light conditions. Under dark condition, the nuclei are 8.0 ± 3 in the ∆*AocryA* mutants compared with 7.0 ± 2 nuclei in the WT strain, and the nuclei are 12.0 ± 3 in the ∆*AocryA* mutants compared with 9.0 ± 3 nuclei in the WT strain under light conditions (Figure 2E,F).

### 3.4. AoCryA Negatively Regulates Trap Formation and Pathogenicity

**Figure 3:**
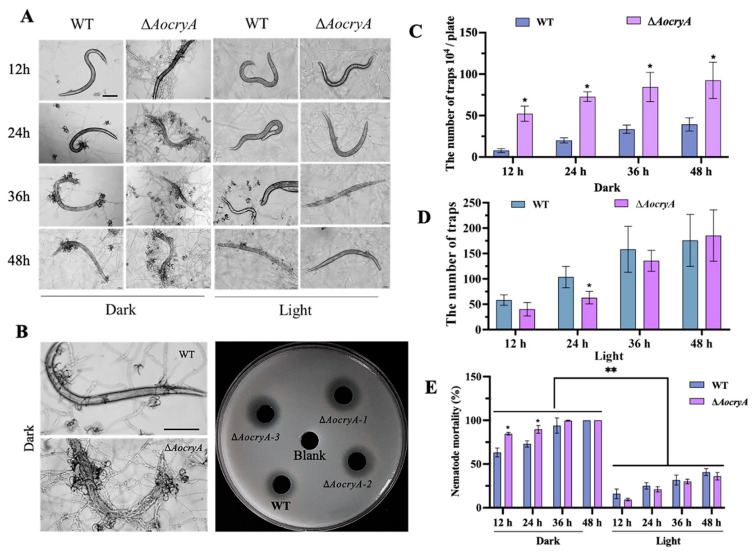
Observations of trap-related traits in WT and ∆*AocryA* mutant strains. (**A**) Trap production under dark and light conditions. Bar—100 µm. (**B**) Degradation of nematodes at 36 h post-induction and qualitative analysis of extracellular proteolytic activity. (**C**) Trap numbers for WT and ∆*AocryA* strains under dark conditions. * *p* < 0.05. (**D**) Trap numbers for WT and ∆*AocryA* strains under light conditions. * *p* < 0.05. (**E**) Nematode mortalities under dark and light conditions. * *p* < 0.05, ** *p* < 0.01.

To characterize the influence of *AocryA* deletion on trap-production and nematode-trapping abilities, the traps were induced by adding 200 *C. elegans* to WA. The morphologies of the traps and the nematode predation abilities of the WT and ∆*AocryA* strains were then observed. The results indicate that the trap-production and nematode-trapping abilities of both WT and mutant strains are influenced by light conditions. In darkness, at 12 h after nematode induction, the ∆*AocryA* mutants formed numerous mature traps, but the WT strain only produced a few immature traps, with single and unclosed trap rings often displayed. Mature traps in the WT strain did not appear until 24 h post-induction (Figure 3A,B). Meanwhile, nearly 90% of nematodes were captured by the ∆*AocryA* strain compared with approximately 70% by the WT strain (Figure 3E). Additionally, at 36 h, the nematodes were degraded in the ∆*AocryA* strain, whereas the nematode cuticle remained distinct in the WT strain, and the extracellular hydrolytic activity of the mutant strain was increased relative to the WT strain (Figure 3B). Under light conditions, the trap production of WT and mutant strains was remarkably decreased compared with dark conditions, and the number of traps of the ∆*AocryA* strain decreased between 12 and 36 h; at the same time, the nematode predation ability of the ∆*AocryA* strain also decreased compared with the WT strain (Figure 3A,D,E).

### 3.5. AoCryA Regulates Stress Response and Carotenoid Synthesis

**Figure 4:**
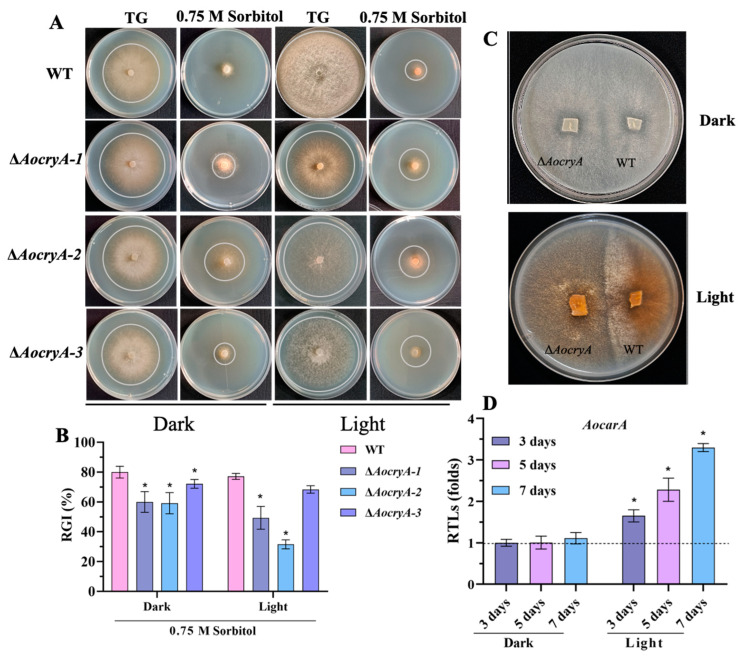
Comparison of sensitivity to osmotic stress and carotenoid synthesis under different light conditions. (**A**) Growth of strains on 0.75 M sorbitol medium under light and dark conditions. (**B**) RGI values of WT and ∆*AocryA* strains under light and dark conditions. * *p* < 0.05. (**C**) Comparison of pigment under light and dark conditions. (**D**) RTLs of a carotenoid synthesis gene (*AocarA*) under light and dark conditions in ∆*AocryA* strains. * *p* < 0.05.

To investigate the sensitivity of the ∆*AocryA* strain to various stressors, the RGI values of the WT and mutant strains were measured under various stressed media. These tests were conducted under both light and dark conditions. Our findings indicate that the mutant strain exhibited increased resistance to osmotic stress (0.75 M sorbitol) under both light and dark conditions compared with the WT strain (Figure 4A,B). Similarly, the mutant strain demonstrated higher resistance to oxidative stress (0.09 M menadione), particularly in dark conditions (Figure S4A,B). However, no obvious differences in RGI values were found between the WT and mutant strains after exposure to cell–wall synthesis interference reagents, such as Congo red and SDS (Figure S4C,D). In addition, the colonies of WT and Δ*AocryA* strains on the PDA medium became brown under light conditions, especially in the Δ*AocryA* mutant (Figure 4C). We also detected transcription of a carotenoid synthesis gene (*AocarA*) in the Δ*AocryA* strain and found that transcription of *AocarA* was remarkably upregulated in the Δ*AocryA* strain after 3, 5, and 7 days under light conditions (Figure 4D).

### 3.6. AoCryA Regulates LD Accumulation and Endocytosis

**Figure 5:**
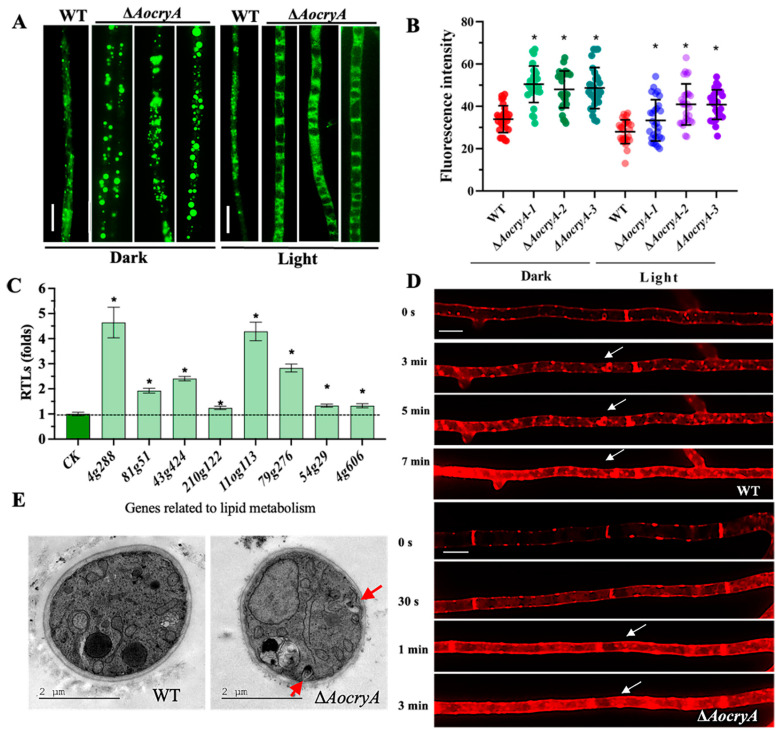
Observation of lipid droplet (LD) accumulation and endocytosis. (**A**) Visualization of LDs in mycelia stained with boron dipyrrolidine dye (BODIPY). Bar—5 µm (**B**) Quantification of LD fluorescence intensity from 50 randomly selected images. * *p* < 0.05. (**C**) RTLs of genes involved in lipid metabolism in the ∆*AocryA* strain versus the WT strain under dark conditions. * *p* < 0.05. (**D**) Visualization of endocytosis in mycelia stained with FM4-64. Bar—5 µm. White arrows indicate the stained membrane structures. (**E**) TEM images showing endocytic vesicles. Red arrows indicate endocytic vesicles.

The WT and ∆*AocryA* strains were cultured on PDA for 5–7 days, and lipid droplets were then stained with BODIPY. Our results reveal that the ∆*AocryA* strain exhibited increased lipid droplet accumulation in dark conditions, a result which was confirmed by a corresponding increase in fluorescence intensity (Figure 5A,B). Furthermore, under dark conditions, the Δ*AocryA* strain displayed rounded LDs; in contrast, under light conditions, the LDs underwent a morphological change, forming dispersive states (Figure 5A,B). The transcription of eight genes involved in lipid metabolism was detected, and their RTLs were upregulated in the Δ*AocryA* strain; these included 3-oxoacyl-[acyl-carrier protein] reductase (AOL_s00004g288), acyl-CoA dehydrogenase (AOL_s00079g276), and 3-hydroxybutyryl-CoA dehydrogenase (AOL_s00110g113) (Figure 5C). Additionally, after staining with FM4-64 for 3 min, the dye was internalized in the mycelia of the Δ*AocryA* mutant, and many membrane structures were stained, but in the WT strain, it remained at the cell surface, and few membranes were stained (Figure 5D). Finally, using TEM, we observed increased numbers of endocytic vesicles in the mycelial cells of Δ*AocryA* mutant compared with the WT strain (Figure 5E).

### 3.7. AoCryA Regulates Secondary Metabolites

**Figure 6:**
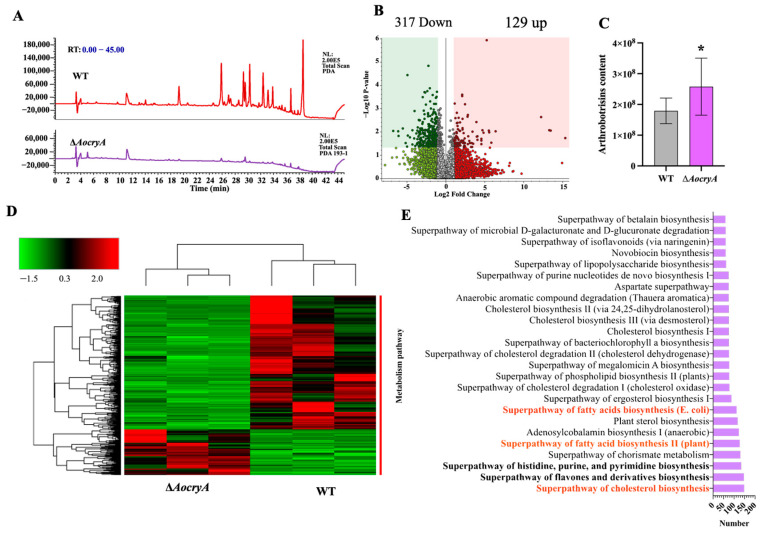
Secondary metabolic analysis of ∆*AocryA* and WT strains. (**A**) Metabolic profiles of the WT and ∆*AocryA* strains. (**B**) Volcano plot analysis of differential expression compounds (DECs). (C) Peak area of arthrobotrisins in the fermentation broth. * *p* < 0.05. (**D**) Cluster analyses of DECs in the ∆*AocryA* mutant and WT strains. (**E**) The top 25 metabolic pathways associated with DECs. Red font indicates partial pathways involved in lipid metabolism. Bold font indicates pathways with the number of genes in the top three.

According to the HPLC profile, the compounds were substantially decreased in the ∆*AocryA* strain compared with the WT strain, especially at appearance times between 26 and 38 min (Figure 6A). Analysis of differential expression compounds (DECs) shows that most metabolites were downregulated in the ∆*AocryA* mutant compared with the WT strain, with 317 compounds downregulated and 129 compounds upregulated (Figure 6B,D). In addition, the content levels of arthrobotrisins, which are produced by *A. oligospora*, a closely related species of NT fungi, were remarkably increased in the ∆*AocryA* strain compared with the WT strain (Figure 6C). According to the enrichment analysis, the top three enriched metabolic pathways for DECs are cholesterol biosynthesis, flavone and derivative biosynthesis, and the superpathway of histidine, purine, and pyrimidine biosynthesis, which contain 148, 147, and 134 DECs, respectively. In addition, several pathways involved in lipid metabolism were enriched, such as the biosynthesis of fatty acids, cholesterol, ergosterol, and phospholipids (Figure 6E).

## 4. Discussion

Previous studies have found that the process of conidia formation involves a number of common developmental pathways, including the temporal and spatial regulation of gene expression, cellular specialization, and intercellular communication, as well as the regulation of environmental factors [46,57]. Many fungi generate spores for transmission, and light is a credible source of information indicating air exposure, which is necessary to initiate spore formation in some fungi [27,37]. A growing body of research into model fungi, such as *A. nidulans, N. crassa*, and *A. fumigatus*, has revealed a variety of sporulation-related regulatory genes in these filamentous fungi [15,39,58,59,60]. Among these, the cryptochrome CryA is a photolytic enzyme that senses blue light [28]. Homologs of CryA are present in broad filamentous fungi, and their sequences are highly similar. In the present study, we identified the function of a homologous CryA (*AocryA*) in *A. oligospora*. Our analysis shows that it plays a key role in mycelial development, sporulation, and trap formation.

Light regulation can lead to changes in light-dependent transcription and subsequent changes in the amount of proteins required for biological reactions [61]. In the present study, the disruption of *AocryA* was found to have no impact on mycelial growth under dark, white-light, and blue-light conditions; the ∆*AocryA* strain showed increased mycelial cell lengths under dark conditions but reduced mycelial cell lengths under white-light conditions compared with the WT strain; and the ∆*AocryA* strains showed greater numbers of nuclei under dark and light conditions, demonstrating that AoCryA plays a role in mycelial development in *A. oligospora*. Under dark conditions, the knockout of *AocryA* caused a remarkable reduction in conidia yield; in contrast, the conidia yield of the WT strain decreased remarkably in the light and exhibited no remarkable change relative to the knockout strain. Correspondingly, under dark conditions, the inactivation of *AocryA* led to a substantial reduction in the transcriptional levels of sporulation-related genes, such as *AoabaA*, *AobrlA*, *AowetA*, *AoflbD*, *AolreA*, and *AolreB* (Figure 2C). It has been shown that *AoabaA*, *AobrlA*, and *AowetA* are essential to conidiation and that they play pleiotropic roles in mycelial development, trap formation, and secondary metabolism [46]. *AolreA* and *AolreB* encode the homologs of, respectively, WC-1 and WC-2, which are the major regulators of blue-light sensing in filamentous fungi [61]. In *A. nidulans*, the spore yields of the Δ*lreA* and Δl*reB* strains increased in dark conditions compared with the WT strain, while the conidia yield of the Δ*fphA* strain decreased. After the addition of white-light irradiation, the trend did not change, with a significant increase in the overall number of conditions compared to dark conditions [24]. In contrast, in *Alternaria alternata*, the numbers of conidia in Δ*lreA* and Δ*fphA* strains were reduced in dark conditions compared with the WT strain. This trend was unchanged by the presence of added light, but the overall number of conidia was significantly lower [62]. These findings demonstrate that light is responsible for divergent regulatory functions on conidial production in different fungi.

In addition, increased pigment was produced in the ∆*AocryA* mutant after exposure to light, as well as an increased expression of the carotenoid synthesis gene. Carotenoid synthesis is a response to light, and the increased accumulation of light-regulated mRNAs (including those for enzymes required for carotenoid biosynthesis) has been found in many fungal studies, in which colonies have been found to take on a dark-brown coloration. For example, in *N. crassa*, the knockout of the protein vivid results in an increase in carotenoids [21,61]. This is consistent with studies of the oleaginous yeast *Rhodosporidium toruloides*, in which transcriptome analysis was used to show that light promotes the transcription of genes related to carotenoid biosynthesis [63,64]. Such results suggest that CryA plays a crucial role in the carotenoid synthesis of *A. oligospora* and other fungi.

The photoreceptor pathway may also influence the virulence of pathogenic fungi [65]. In the present study, under dark culture conditions, the knockdown of *AocryA* resulted in an approximately six-fold increase in the number of traps, as well as a significant increase in nematode-trapping ability. In contrast, trap formation and nematode-trapping ability were substantially reduced in the light. In the yeast *Cryptococcus neoformans* and the ascomycete fungus *Fusarium oxysporum*, deletion of the direct homolog of *wc-1* resulted in diminished virulence in a mouse model of infection. Furthermore, light has been shown to have a profound effect on the virulence of human pathogens in an epithelial infection model [66], although the response is not the same in different species: virulence was enhanced by light in *Acinetobacter baumannii* and *Staphylococcus aureus*, while in *Acinetobacter nosocomialis* and *Pseudomonas aeruginosa,* it was reduced [67]. Therefore, light is an important environmental factor driving the expression of virulence in these pathogens.

Fungi can adapt to conditions of stress by sensing light signals. A link between light and stress-signaling has been found in a number of fungi [21]. In addition, the environmental adaptation and sensitivity of nematophagous fungi are closely related to potential roles in biological control [43]. In the present study, the ∆*AocryA* strain was found to be less sensitive to high concentrations of sorbitol under both light and dark conditions. This result is consistent with the finding of a previous study on *A. nidulans*, namely, that other photoreceptors, such as FphA, sense red-light signals and then initiate the HOG pathway via the phosphotransferase protein YpdA to enhance resistance to hyperosmolarity [35]. In contrast, mycelial growth is not obviously inhibited by stresses from cell wall-disturbing and oxidative reagents. We may say, then, that homologs of CryA play a conserved role in sensing light and regulating osmotic stress.

Energy metabolism is essential to fungal growth and development and pathogenicity [40]. LDs are storage organelles whose biogenesis is closely linked to cellular metabolism and which are essential for buffering levels of toxic lipid substances [68]. In the ∆*AocryA* mutant, the volume of LDs was remarkably enlarged, and the expression of lipid metabolism-related genes was upregulated. Enzymes such as 3-oxoacyl-[acyl-carrier protein] reductase, acyl-CoA dehydrogenase, and 3-hydroxybutyryl-CoA dehydrogenase are involved in the beta-oxidation of fatty acids [69]. Under white light, the LDs become dispersed under light irradiation. Interestingly, our metabolomic analysis reveals that several DECs were enriched in pathways associated with lipid metabolism, such as the biosynthesis of fatty acids, cholesterol, ergosterol, and phospholipids. These results indicate that AoCryA is required for LD accumulation and plays a role in lipid metabolism. In addition, *AocryA* affects endocytosis. After staining with FM4-64, we found that the mycelium of the WT strain did not fully internalize the dye into the cytoplasm and vesicles after 3 min of treatment. In contrast, FM4-64 rapidly entered the mycelium of the ∆*AocryA* strain. Additionally, more phagocytic vesicles were found in the ∆*AocryA* strain compared with the WT strain, a result which further indicated the absence of *AocryA*-enhanced endocytosis. Our previous studies show that multiple proteins are involved in the regulation of mycelial growth, LD accumulation, conidiation, and trap formation, such as SNARE protein AoSec22 [45], malate dehydrogenase AoMae1 [47], and response regulator AoSsk1 [59], and their results suggest that mycelial growth and development are very complicated and controlled by diverse cellular processes.

In addition, light plays an essential role in the secondary metabolism of filamentous fungi. In *A. nidulans*, partial loss-of-function mutations in the light-responsive velvet gene *veA* can mediate extensive alterations in secondary metabolism [22,70]. Light also has a wide range of effects on the metabolism of *A. fumigatus* [65]. In this study, the inactivation of *AocryA* caused changes in secondary metabolites, including arthrobotrisins. Moreover, several pathways involved in lipid metabolism were remarkably enriched, consistent with the accumulation of LDs in the ∆*AocryA* mutant. We may say, therefore, that the function of homologous CryA is conserved in the secondary metabolism of filamentous fungi and that AoCryA plays an important role in LD accumulation and trap formation in *A. oligospora*.

## 5. Conclusions

The results of the present study demonstrate that the cryptochrome CryA is critical for regulating the mycelial development, conidiation, and pathogenicity of *A. oligospora*. AoCryA is crucial for spore formation and is involved in endocytosis, LD accumulation, osmotic stress response, and secondary metabolism. Importantly, AoCryA negatively regulates trap formation and nematode-trapping ability. Under light exposure, the ∆*AocryA* mutant was found to exhibit increased mycelial growth and an increase in carotenoid biosynthesis. This study is the first to reveal the role of CryA in the mycelial development, conidiation, and trap-formation ability of *A. oligospora*. Additionally, we established a connection between light exposure and sporulation and trap formation in NT fungi, and this may help future researchers to reveal the mechanism by which light signaling regulates fungal development and pathogenicity.

## Data Availability

Data are contained within the article and Supplementary Materials.

## Supplementary Materials

The following supporting information can be downloaded at: https://www.mdpi.com/article/10.3390/jof10090626/s1, Figure S1: Plasmids pRS426 and pCSN44 were mapped by SnapGene (Version 6.0.2). Figure S2: Phylogenetic analysis and validation of ∆*AocryA* knockout strain. Figure S3: Mycelial growth rates of the WT and the ∆*AocryA* strains on TG and TYGA media. Figure S4: Comparison of the sensitivity of ∆*AocryA* and WT strains to stressors; Table S1: List of primers used for gene manipulation and RT-PCR analysis in this study.
