# Supplementary material for: The Cryptochrome CryA Regulates Lipid Droplet Accumulation, Conidiation, and Trap Formation via Responses to Light in Arthrobotrys oligospora
Source: J Fungi (Basel). 2024 Sep 1;10(9):626. doi: 10.3390/jof10090626 (PMC11432822; doi:10.3390/jof10090626)
Supplement: Supplementary file 1 [file jof-10-00626-s001.zip › jof-3142287-supplementary.pdf]

Supporting Information

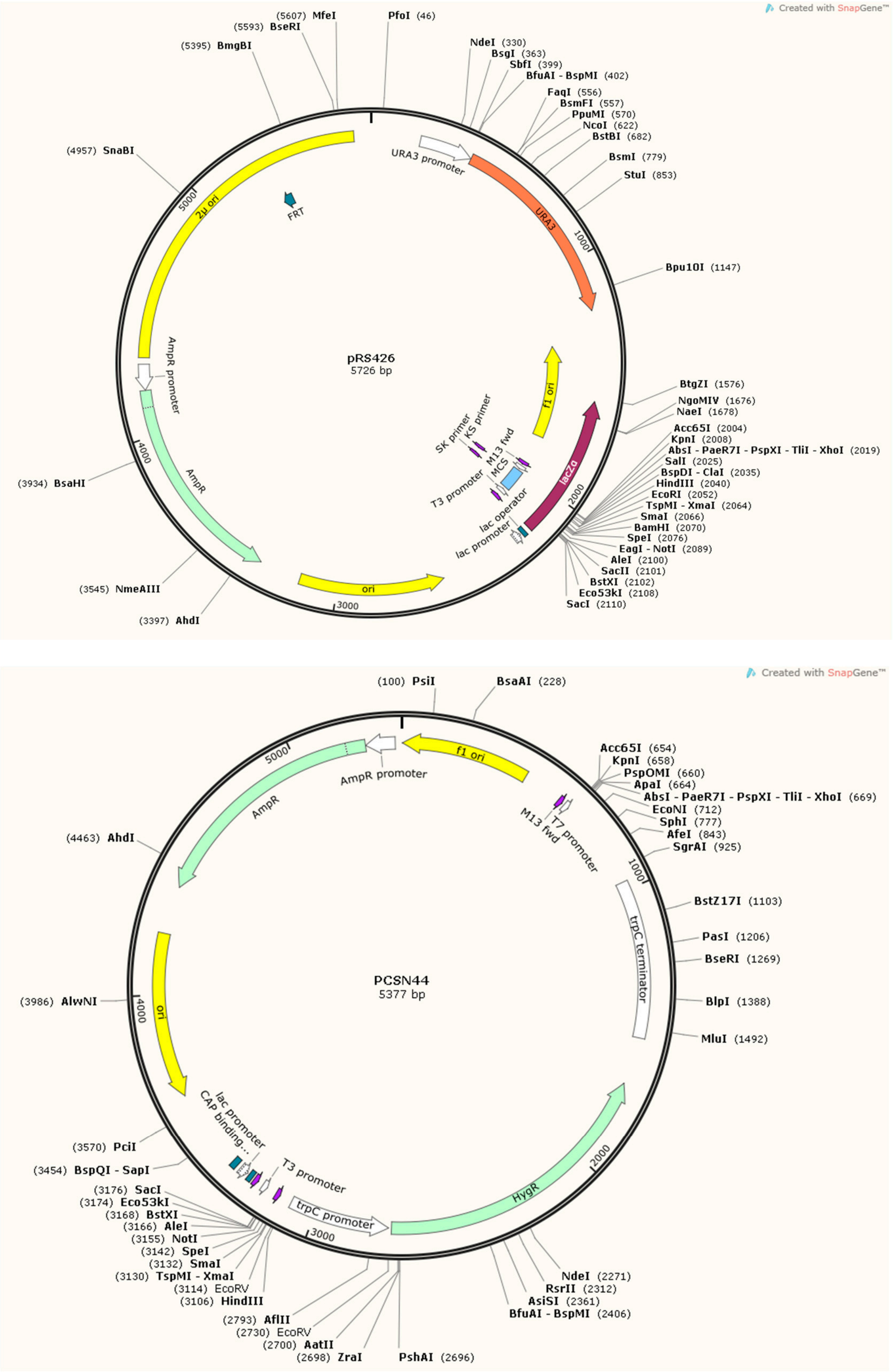

Figure S1. Plasmids pRS426 and pCSN44 were mapped by SnapGene (Version 6.0.2). The information of plasmids pRS426 and pCSN44 was referred to references Staben et al. (1989) and Christianson et al. (1992).

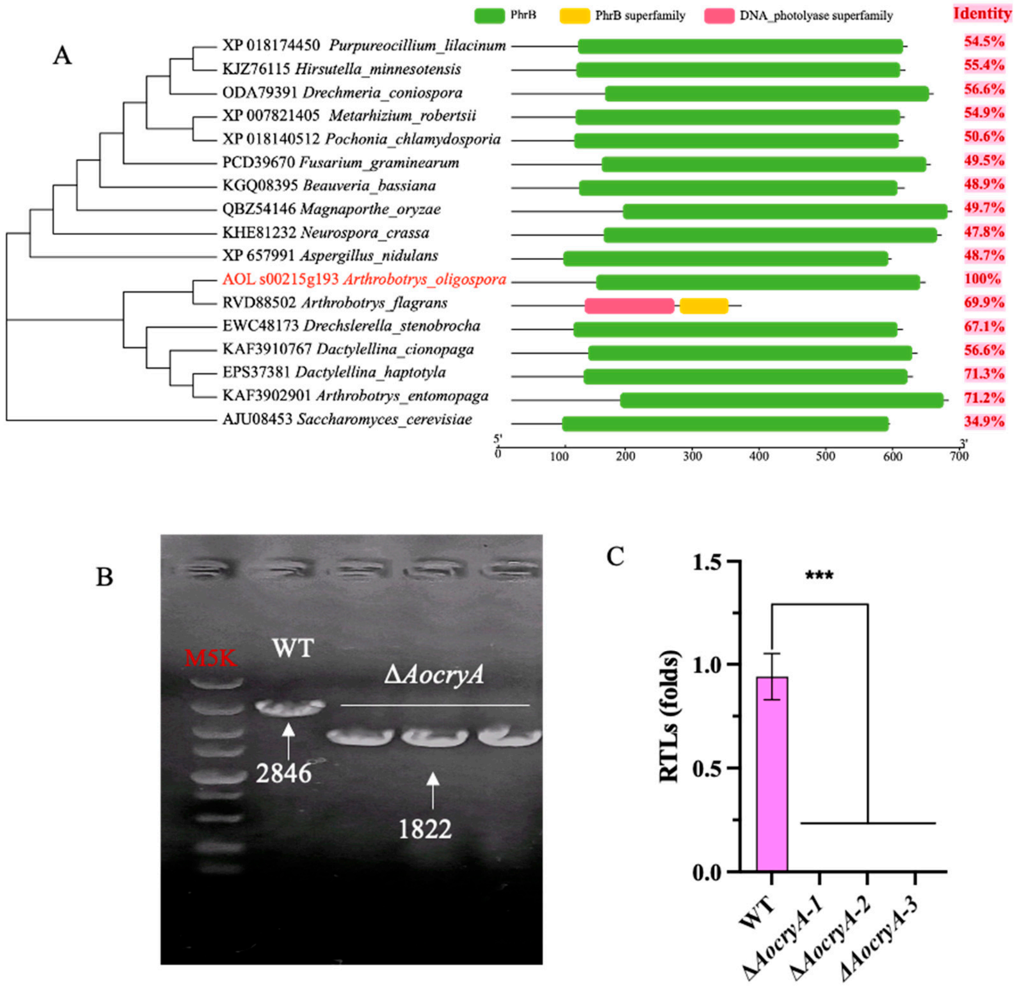

Figure S2. Phylogenetic analysis and validation of *AocryA* knockout strain. A Phylogenetic and structure domain analysis of CryA orthologs from different fungi. (B and C) Validation of knockout strains by PCR (B) and RT-PCR (C). M2K, DNA marker. An asterisk indicates a significant difference between the Δ*AocryA* mutant and the WT strain (Tukey's HSD, **p* < 0.01).

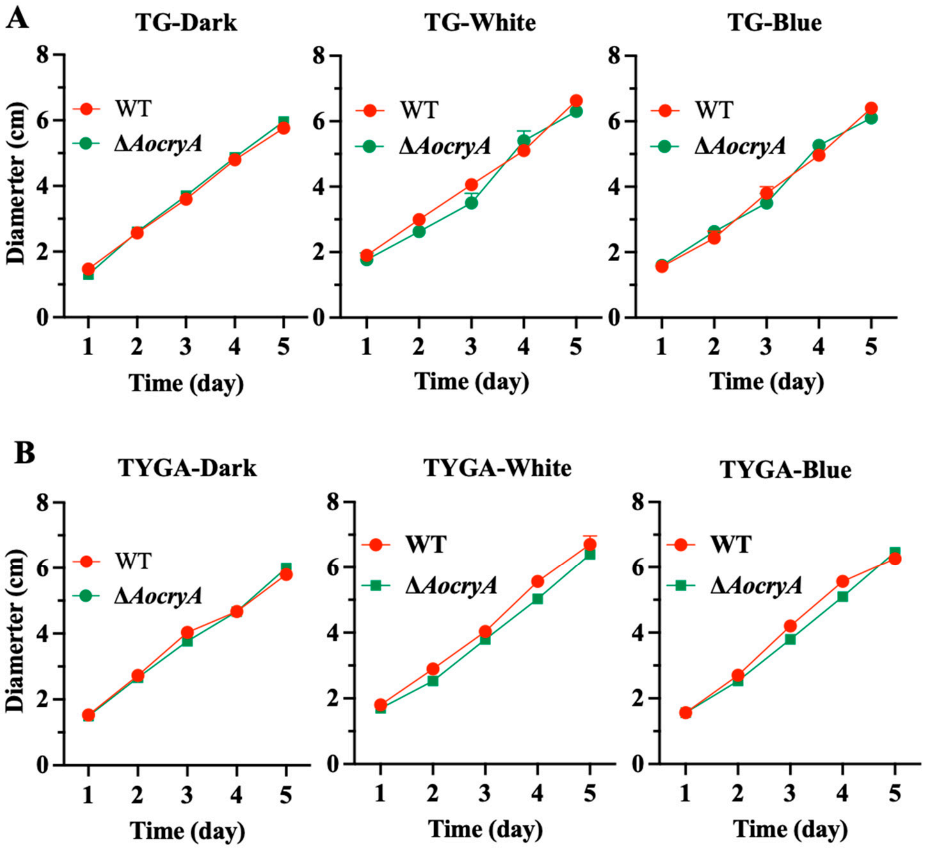

Figure S3. Mycelial growth rates of the WT and the Δ*AocryA* strains on TG and TYGA media. (A) The comparison of colony growth rates under different light conditions on TG medium. (B) The comparison of colony growth rates under different light conditions on TYGA medium.

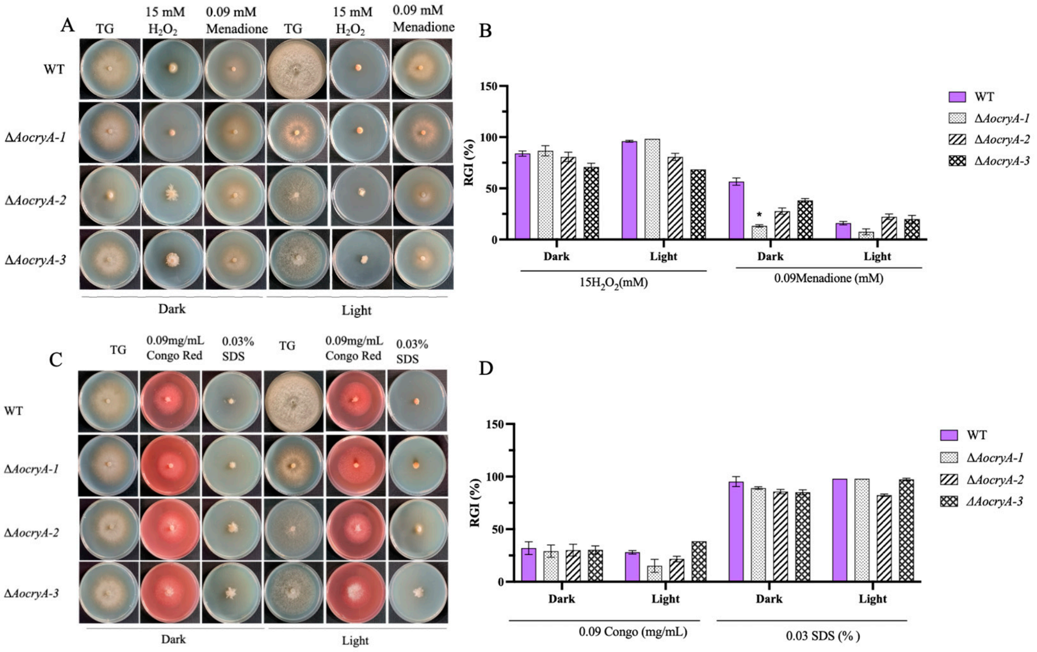

Figure S4. Comparison of the sensitivity of Δ*AocryA* and WT strains to stress. (A) Growth of strains on 15 mM H_2O_2 medium and 0.09 mM menadione medium in light and dark. (B) Relative growth inhibition (RGI) of WT and Δ*AocryA* mutant strains by H_2O_2 and menadione under different conditions. (C) Growth of strains on 0.09 mg/mL Congo red medium and 0.03% SDS medium in light and dark. (D) Relative growth inhibition (RGI) of WT and Δ*AocryA* mutant strains by Congo red and SDS under different conditions, $^*p < 0.05$.

Table S1. List of primers used for gene manipulation and RT-PCR analysis in this study.

Primers	Sequences	Application
AocryA-5F	GTAACGCCAGGGTTTTCCCAGTCACGACGATGCTCGGGTCCACTTCG	Amplify the *AocryA* gene 5’ flank
AocryA-5R	ATCCACTTAACGTTACTGAAATCTCCAACGCTTTGACAACCTGCTCCTT	
AocryA-3F	CTCCTTCAATATCATCTTCTGTCTCCGACTAGAGGTCGAACTCAGGAGCC	Amplify the *AocryA* gene 3’ flank
AocryA-3R	GCGGATAACAATTTCACACAGGAAACAGCCCACCAAGTGCGAGAAAGAA	
Hph-f	GTCGGAGACAGAAGATGATATTGAAGGAGC	Amplify the hph cassette
Hph-r	GTTGGAGATTTCAGTAACGTTAAGTGGAT	
CyA-PF	CAGCATACGGTCCCTCAA	Primers for PCR verification
CyA -PR	ACATGGGCAATTCAAAG	
CyA -RF	AAATCCTGGGAGCCGTATGTTT	Primers for RT-qPCR verification
CyA -RR	GGAGTGGGGAAAGAAGTCTGAG	
β-tubulin gene	Sequence (5′-3′)	
AOL_s00076g640 (*tub*)	tubA-F-CCACCTTCGTCGGTAACTC	
	tubA-R-TCGTCCATACCCTCACCAG	
Sporulation-related genes	Sequence (5′-3′)	
AOL_s00080g63 (*abaA*)	63-5F-AACTTTATGCGCCTTGTCGT	
	63-3R-TTGGCTAGGTGGTCTGTACG	
AOL_s00210g120 (*medA*)	120-5F-TCCGGCCCAATGATTCAGAA	
	120-3R-AGATCGCAGGAACATGGTGA	
AOL_s00097g46 (*flbD*)	46-5F-TGACCCAATCACACCAGAGG	
	46-3R-CGCTCCTGTTCTACCGATGA	
AOL_s00097g514 (*brlA*)	514-5F-TTGAGGCCTCGATCCGTAGA	
	514-3R-AGGTAGATGGCGCTGTTACG	
AOL_s00173g221 (*wetA*)	221-5F- TTACATGCCACCCCAAGTCC	
	221-3R- CAATTGCAACTGCGTCCACA	
AOL_s00083g487 (*lreA*)	487-5F-TTCTCTTCGTCCCAAGCCAC	
	487-3R-ACCGGTTCGAGTGGAGTCTA	
AOL_s00080g93 (*lreB*)	93-5F-CCAGGGTCGTCAGTATCTT	
	93-3R-CAGCATCTTCCAGGTCAA	
AOL_s00078g317(*flbB*)	317-5F-AATTCCAGCATCGTTAGTGGGT	
	317-3R-TATCCAACATATGCGACTCCGG	
AOL_s00215g48(*fphA*)	48-5F-CACCTCCAGCAGAAAATACCCT	
	48-3R-AGAACCCGCAATGGCTTACTAA	
Lipid metabolism-related genes	Sequence (5′-3′)	
AOL_s00004g288	288-5F-AAGAAATCCCACTTCAGAGAGG	
	288-3R-TACGTGTCCAGTAACATAGCTC	
AOL_s00081g51	51-5F-GCCGATCCTTACCAAATCATTC	
	51-3R-CCAATTCTTTTCCGTAGCTGAG	
AOL_s00043g424	424-5F-CTCTGCTCTATGGATACGAACA	
	424-3R-AGATGAACTTCTCGACTTCTCC	
AOL_s000210g122	122-5F-GCCGCACATATTGTTAACAGAT	
	122-3R-TGATCTTGCTGTTCTCAGTCAT	
AOL_s000110g113	-5F-CTAACAGAAACTCAAGCATCGG	
	-3R-GGAACCGGATTCATGAAATGAG	
Carotenoid synthesis genes	Sequence (5′-3′)	
AOL_s00004g586 (*carA*)	586-5F-TCCTTCACCTTGTTTTCCACCA	
	586-3R-TGTATTCTGCCAGCCAATCACT	

Light regulation-related genes	Sequence (5′-3′)
AOL_s00007g63 (*conj*)	63-5F-TTCGAAAGTCATAAGCCCGTCA
	63-3R-GCCTCGGCAAAAAGGTATTTGT
AOL_s00083g316 (*vvd*)	316-5F-TTGTGATGTCCATGACTCTGGG
	316-3R- CTGGGAATGTCCACCATGAGAA
AOL_s00054g216 (*ccg-8*)	216 -5F-AAATGGTACCAGCGGGAAAGAT
	216 -3R-TGACCACTACCCTTGCATTCTC
AOL_s00054g633 (*frq*)	633-5F-GGATTCCGAAGATGAGAGCGAT
	633-3R- TCATAGACTTTGCCAGCACCAT
